# Pearls of Elschnig

**DOI:** 10.18502/jovr.v14i4.5469

**Published:** 2019-10-24

**Authors:** Brian K Foutch, Charles A Garcia, Amy S Ferguson

**Affiliations:** ^1^University of the Incarnate Word, Rosenberg School of Optometry, TX, USA

##  PRESENTATION

A 72-year-old Hispanic male patient presented with a chief complaint of blurred near vision without spectacles. He had a medical history of hypercholesterolemia, hypertension, and type 2 diabetes mellitus, controlled with medication; an ocular history of chronic uveitis about three decades ago; and a history of cataract surgery and posterior-chamber intraocular lens (PCIOL) implantation in the left eye seven years back.

The patient's visual acuity was correctable to 20/20 in each eye with refraction (bilateral compound hyperopic astigmatism). Extraocular motilities were unrestricted, and confrontation fields were full to finger count. The pupils were reactive to light and no afferent pupil defect (APD) was present. The right pupil was 4 mm and round in dim light, but the left one had a slight vertical elongation, commonly seen in an operated eye.

**Figure 1:**
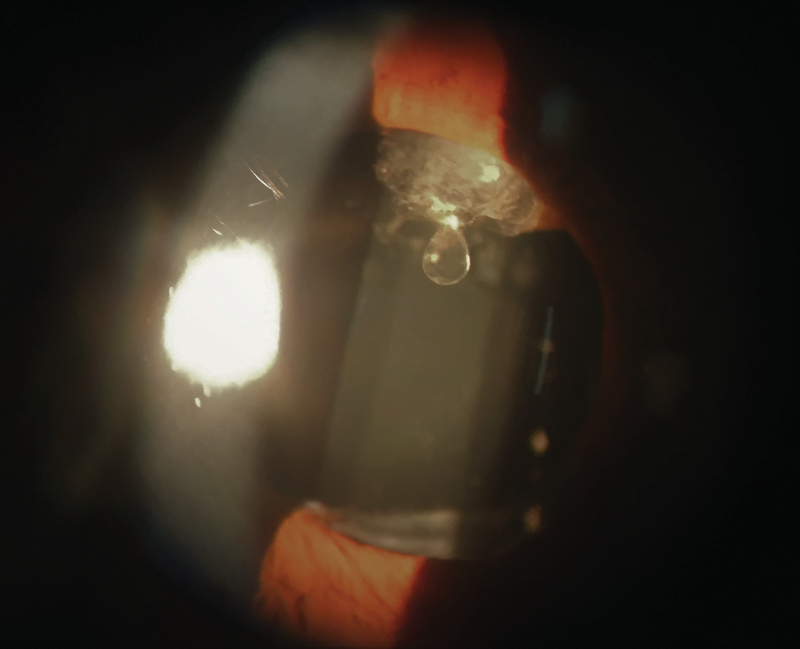
Biomicroscopic photograph showing the Elschnig pearls in the left eye. This original image was captured using an iPhone 6 camera mounted with an adapter to a Topcon® slit lamp biomicroscope (Topcon Medical Systems Inc., Oakland NJ). (Tiger Lens is unavailable to provide any information to me other than the website.)

The anterior segment examination revealed deep and quiet anterior chambers; the right iris and lens appeared normal with grade 1${}^{+}$ lenticular nuclear sclerosis (NS). The PCIOL appeared stable and centered in the left eye, but there was a large grape-like mass postero-superiorly to the iris [Figure 1].

**Figure 2:**
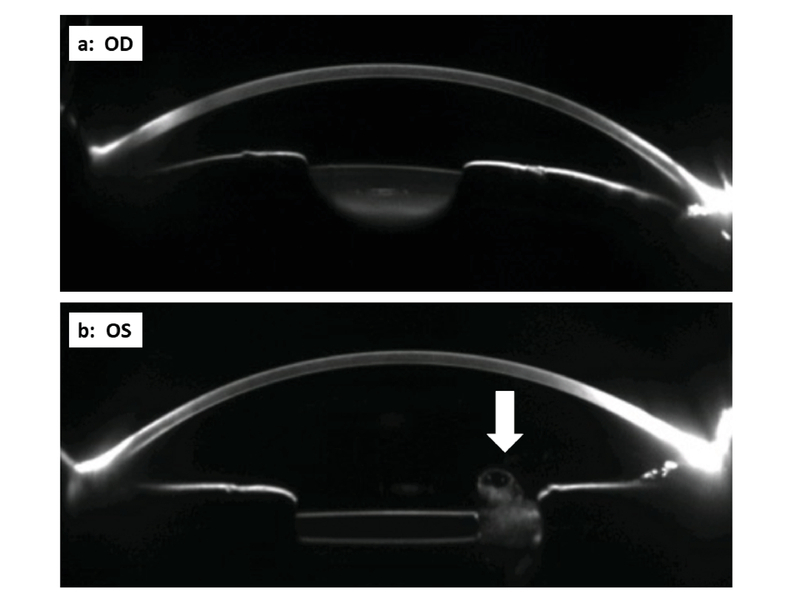
Scheimpflug anterior segment reconstructions of right eye (top) and left eye (bottom) captured in the clinic. The large grouping of Elschnig pearls is easily seen in the bottom image.

Dilated funduscopic examination revealed healthy optic nerves with normal cupping in both eyes. The maculae were flat and all vessels appeared healthy. The peripheral retinal examination of OD revealed a small (∼1 mm) horseshoe tear without traction infero-temporally but was unremarkable in OS. The patient was referred for a retinal consultation for the retinal tear in the right eye. Additional assessments showed type 2 diabetes mellitus without retinopathy, pseudophakia with an unusually large presentation of Elschnig pearls in OS.

Scheimpflug anterior segment reconstructions were performed using PentacamⓇ (OCULUS Inc., Arlington WA); the iris-lens configurations generated for both eyes are shown in Figure 2. The NS of the right eye is shown in Figure 2(a), and Figure 2(b) shows the mass of cells clumped like a jewel and extending into the anterior chamber. The patient was provided with a new spectacle prescription and instructed to return to the clinic after a year.

##  DISCUSSION

Elschnig pearls were once considered to be a rare complication of cataract surgery,^[1]^ and they can be difficult to distinguish epidemiologically from other forms of posterior capsular opacification (PCO). Hence, their precise incidence may not have been correctly reported.^[2]^ There is a small body of literature on the pathogenesis and management of Elschnig pearls,^[1,2,3,4,5]^ and this report may be the first showing a dramatic presentation without any effect on visual acuity.

It is conceivable that the impressive size of these pearls is due to a lack of posterior sub-capsular scaffolding to contain the spread of the epithelial cells as seen in typical cases. The nature of this case may have also been influenced by the patient's history of uveitis, or its treatment, 30 years ago.^[3]^ However, we are unaware of any specific postoperative history, and it has been reported that individual variability, rather than inflammation, has the greatest effect on the formation of Elschnig pearls.^[4]^

##  Financial Support and Sponsorship

Nil.

##  Conflicts of Interest

There are no conflicts of interest.
